# Expression of p53, p63, podoplanin and Ki-67 in recurring versus non-recurring oral leukoplakia

**DOI:** 10.1038/s41598-021-99326-5

**Published:** 2021-10-21

**Authors:** Jonas Sundberg, Sushma Pandey, Daniel Giglio, Erik Holmberg, Göran Kjeller, Anikó Kovács, Lars Peter Sand, Burcu Tokozlu, Jenny Öhman, Dipak Sapkota, Bengt Hasséus

**Affiliations:** 1 Department of Oral Medicine and Pathology, Institute of Odontology, The Sahlgrenska Academy, University of Gothenburg, P.O. Box 450, 40530 Gothenburg, Sweden; 2 Department of Oral Biology, University of Oslo, Oslo, Norway; 3 Department of Oncology, Institute of Clinical Sciences, The Sahlgrenska Academy, University of Gothenburg, Gothenburg, Sweden; 4 Department of Oral and Maxillofacial Surgery, Institute of Odontology, The Sahlgrenska Academy, University of Gothenburg, Gothenburg, Sweden; 5 Department of Clinical Pathology, Sahlgrenska University Hospital, Gothenburg, Sweden; 6 Department of Oral Pathology, Faculty of Dentistry, Gazi University, Ankara, Turkey; 7 Clinic of Oral Medicine, Public Dental Service, Region Västra Götaland, Gothenburg, Sweden

**Keywords:** Oral cancer, Oral cancer detection

## Abstract

Oral leukoplakia (OL), a potentially malignant disorder, recurs in 40% of cases after surgical removal. Recurrence is a risk factor for malignant transformation. We aimed to examine the prognostic significance of four biomarkers related to cell proliferation: p53, p63, podoplanin (PDPN) and Ki-67 in predicting recurrence. Formalin-fixed-paraffin-embedded specimens from excised OL (n = 73, 33 recurrent; 40 non-recurrent) were collected in a prospective study. Immunohistochemistry was used to visualise expression of p53, p63, PDPN and Ki-67. Image analysis software was used for quantification of p53-, p63- and Ki-67-expressing cells, while PDPN was analysed visually. The expression of all four proteins were higher in recurrent compared with non-recurrent OL, only expression of p53 was statistically significant. In uni- and multivariable Cox regression analyses of individual markers, expression of p63 was significantly associated with higher recurrence risk (*p* = 0.047). OL with a combined high expression of both p53 and p63 had a significantly higher risk to recur [Log Rank, *p* = 0.036; multivariate Cox, HR: 2.48 (1.13–5.44; *p* = 0.024)]. Combination of p53 and p63 expression may be used as a prognostic biomarker for recurrence of OL.

## Introduction

Oral leukoplakia (OL) is an oral potentially malignant disorder (OPMD) and has the potential to transform into oral squamous cell carcinoma (OSCC)^1^. Early detection of OSCC is of great importance since it is associated with an increased survival rate^2^. Standard of care for OL is, if possible, to surgically remove the lesion^3^. Despite surgical removal, recurrence rates range between 10 and 45% depending on the type of lesion and surgical method used^4–6^. Importantly, complete removal of the lesion does not eliminate the risk for future transformation to OSCC^5,7,8^.

Currently, a combination of clinical features and histopathological assessment of presence of dysplasia is gold standard to estimate the risk for malignant transformation and to plan clinical follow-up intervals^3^. One of the main drawbacks of this diagnostic system is considerable inter- and intra-observer variability^9^. Presence of dysplasia is neither sensitive nor specific enough to screen out OL with malignant potency^8^. In parallel, clinical type of OL—homogenous or non-homogenous—does not predict cancer risk with certainty, although the non-homogenous type is associated with a higher risk for transformation to OSCC^10^. Thus, neither clinical type nor dysplasia status is sufficient as sole markers for malignant transformation.

This lack of objectivity in the histopathological assessment has been pointed out and there is a need for reliable biomarkers to risk assess OL. Efforts have been made to reduce the subjectivity of the grading system by investigating the correlation between different biomarkers and malignant transformation. Hence, many biomarkers have been proposed to have a prognostic value for OL^11^. p53, p63, Ki-67 and podoplanin (PDPN; D2-40) are biomarkers that have been proposed to be of importance to identify OL with high risk for transformation to OSCC^11–15^.

“The guardian of the genome”-p53—is a tumour suppressor protein detecting DNA aberrations in cells. p53 is involved in cell cycle control, apoptosis and the preservation of genomic stability^16^. p53 has been extensively studied and has been pointed out as a biomarker for predicting both dysplasia and malignant transformation of OL^12–14^. The *TP63* gene, a homolog of *TP53*, gives rise to two distinct protein isoforms (TAp63 and DNp63) through the utilization of alternative promotors^17^. In general, the TAp63 isoform displays cell proliferation suppressive functions, while the DNp63 is functionally linked to promotion of stemness and proliferative ability of keratinocytes^18^. p63 has been shown to be upregulated in both OSCC and oral dysplastic lesions^14,19,20^. Together, p63 and p53 regulate cell proliferation and differentiation and may play a role in the malignant transformation of OL^21^. Thus, both proteins should be feasible biomarker candidates for recurrence and cancer transformation of OL.

PDPN is a mucin type transmembrane glycoprotein that is widely expressed in different cells and tissues^22,23^. PDPN is upregulated in basal epidermal keratinocytes and dermal fibroblast-like cells under hyperproliferative conditions, such as wound healing, psoriasis or upon inflammatory stimuli^24^. Functional studies have shown that PDPN promotes tumour progression^23^. In OL, PDPN has been demonstrated to be a useful biomarker to assess the risk of malignant transformation and has been shown to be overexpressed in OSCC^25,26^.

The Ki-67 protein is a cell proliferation marker and extensively studied in OL and OSCC. Expression of Ki-67 is correlated with the grade of dysplasia and malignant transformation^27^. Ki-67 is expressed in the G2-M-phase and in the latter half of the S-phase^28^. The Ki-67 expression pattern and intensity have been correlated to both dysplastic oral lesions and to malignant transformation^13,29^.

We, and others, have previously shown that recurrence rates of OL after complete surgical removal are high and that recurrence is a risk factor for malignant transformation^4,30^. The cause of recurrence may be attributed to genetic aberrations involving control of cell proliferation throughout the oral epithelium. This is also in line with the concept of field cancerization, which has been proposed to explain the origin of multiple primary OSCC in some patients^31^.

Thus, we hypothesize that there is a difference in cell proliferation signalling in epithelial cells in recurring vs. non-recurring OL. The aim of this study is to compare expression of four biomarkers related to cell proliferation control: p53, p63, PDPN and Ki-67 in recurring OL (ROL) and non-recurring OL (NOL).

## Results

### Clinical data

Of the 73 OL biopsies, 33 (45%) recurred and 40 (55%) did not (Table 1). Median follow-up time for all patients, including to the end of follow-up for those censored, was 3.1 years (mean: 2.9; range: 0.2–5.7 years). In the ROL group median follow-up time to recurrence was 1.4 years (mean: 1.8; range: 0.2–4.1 years) and in the NOL group 4.3 years (mean: 3.8 range: 1.1–5.7 years). In the ROL group, 14 (48%) of the lesions were homogenous as compared with 29 (73%) in the NOL group. Dysplasia was observed in 8 (24%) and 6 (10%) of ROL and NOL samples, respectively. Twenty (61%) and 19 (48%) were males in the ROL group and NOL group, respectively. Mean age in the ROL group was 60 years and in the NOL group 61 years. In the ROL group 15 (58%) of the OL had a larger area than 200 mm^2^ and in the NOL group 30 (75%) of the OL were larger than 200 mm^2^. Multiple lesions were found in 22 (67%) of the ROL and 19 (48%) in the NOL. Five patients (15%) from the ROL group were identified as smokers, compared with 8 patients (20%) from the NOL group. The use of snuff was seen in 6 (18%) of the patients in the ROL group and 1 (3%) patient in the NOL group. Cancer transformation was seen in 3 patients (9%) with ROL and in none of the NOL patients.

**Table 1:** Clinical characteristics and anamnestic data of the included patients (n = 73).

	No recurrence	Recurrence	Total
	N (%)	N (%)	N (%)
Patients	40 (55)	33 (45)	73 (100)
**Age**			
Mean, median, (range)	61, 62 (35–80)	60, 60 (31–81)	61, 61 (35–81)
**Sex**			
Male	19 (48)	20 (61)	39 (53)
Female	21 (52)	13 (39)	34 (47)
**Site of lesion**			
Floor of the mouth	0 (0)	2 (6)	2 (3)
Buccal mucosa	7 (18)	3 (9)	10 (14)
Lateral tongue	6 (15)	10 (30)	16 (22)
Ventral tongue	5 (13)	1 (3)	6 (8)
Dorsum tongue	0 (0)	1 (3)	1 (1)
Soft palate	1 (3)	0 (0)	1 (1)
Hard palate	5 (13)	3 (9)	8 (11)
Mandibular alveolar gingiva	9 (23)	7 (22)	16 (22)
Maxillary alveolar gingiva	5 (13)	5 (15)	10 (14)
Lip	2 (5)	1 (3)	3 (4)
**Clinical diagnosis**			
Homogenous	29 (73)	14 (48)	43 (59)
Non-homogenous	11 (28)	19 (52)	30 (41)
**Size**			
≥ 200 mm^2^	10 (25)	14 (42)	24 (33)
< 200 mm^2^	30 (75)	19 (58)	49 (67)
**Dysplasia**			
Yes	6 (15)	8 (24)	14 (19)
No	34 (85)	25 (76)	59 (81)
**Multiple lesions**			
Yes	19 (48)	22 (67)	41 (56)
No	21 (52)	11 (33)	32 (44)
**Smokers**			
Yes	8 (20)	5 (15)	13 (18)
No	32 (80)	28 (85)	60 (82)
**Smokers in the past**			
Yes	5 (12)	17 (52)	22 (30)
No	32 (80)	14 (42)	46 (63)
No data	3 (8)	2 (6)	5 (7)
**Snuff users**			
Yes	1 (3)	6 (18)	7 (10)
No	39 (97)	27 (82)	66 (90)
**Snuff users in the past**			
Yes	5 (12)	3 (9)	8 (11)
No	32 (80)	23 (70)	55 (75)
No data	3 (8)	7 (21)	10 (14)
**Alcohol consumption**			
Alcohol daily	0 (0)	1 (3)	1 (1)
Several times per week	4 (10)	4 (12)	8 (11)
Once a week	12 (30)	12 (36)	24 (33)
Rarely/never	19 (48)	13 (40)	32 (44)
Never	0 (0)	0 (0)	0 (0)
No data	5 (12)	3 (9)	8 (11)
**Cancer transformation**			
Yes	0 (0)	3 (9)	3 (4)
No	40 (100)	30 (91)	70 (96)

### p53 expression in non-recurring and recurring leukoplakia

**Figure 1:**
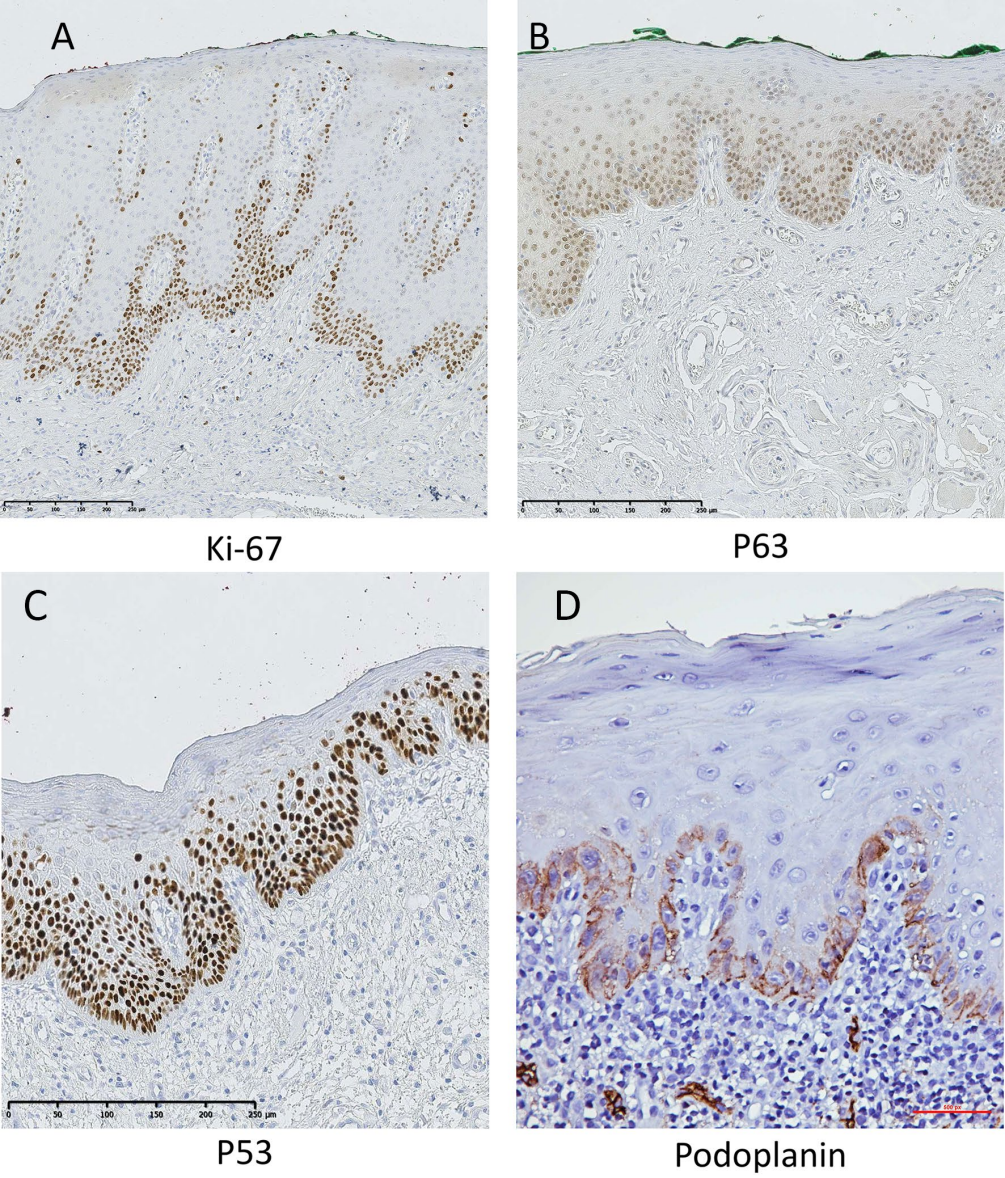
Representative images for immunohistochemical expression of Ki-67 (**A**), p63 (**B**), p53 (**C**) and Podoplanin (**D**) in leukoplakia. QuPath: Open source software for digital pathology image analysis (version 0.2.0-m4)^46^ was used to create the figure.

**Figure 2:**
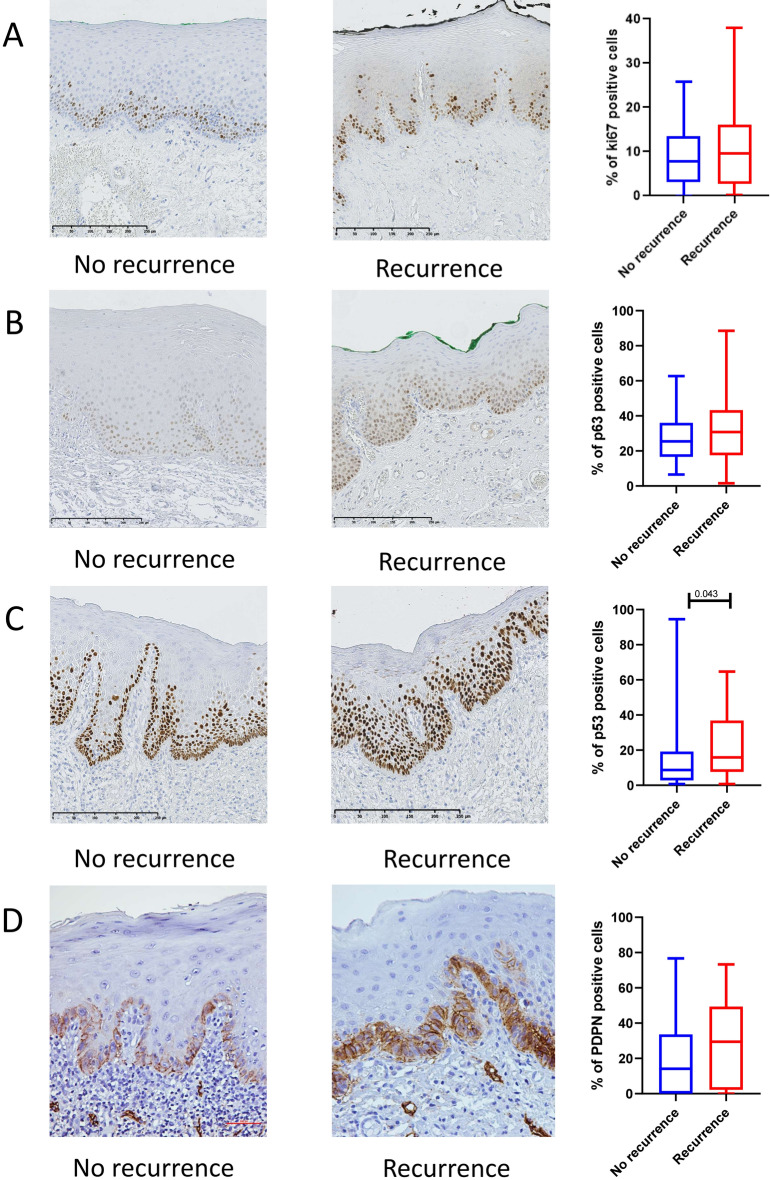
Representative images for the immunoexpression and boxplots for percentage of positive cells in recurring versus non-recurring leukoplakia for Ki-67 (**A**), p63 (**B**), p53 (**C**) and podoplanin (d240) (**D**). Box plot graphs showed median (horizontal bars), interquartile range, and maximum and minimum values (whiskers). QuPath: Open source software for digital pathology image analysis (version 0.2.0-m4)^46^ and Stata Statistical Software (release 16) were used to create the figure.

Nuclear staining of p53 was observed mainly in basal and suprabasal keratinocytes (Fig. 1). The non-recurring OL (NOL) were found to express significantly lower levels of p53 as compared to the ROL (median: 8.5%, mean: 15.7%; range: 1–95 in NOL versus 15.5%, mean: 23.6% and range: 0–38 in ROL (*p* = 0.043; Fig. 2).

### p63 expression in non-recurring and recurring leukoplakia

p63 expression was predominantly detected in nuclei of keratinocytes (Fig. 1). Although the NOL were found to express lower levels of p63 as compared to the ROL (median: 25.0%, mean: 27.7%; range: 7–63 in NOL versus 31.0%, mean: 33.3% and range: 2–86 in ROL), the results were not statistically significant (Fig. 2).

### Podoplanin expression in non-recurring and recurring leukoplakia

PDPN-expressing cells were detected both in epithelium and in endothelium of the lymphatic vessels in the connective tissue (Fig. 1). Although the NOL were found to express lower levels of PDPN in epithelium as compared to the ROL (median: 14.0%, mean: 20.7%; range: 0–77 in NOL versus 29.0%, mean: 28.2% and range: 0–73 in ROL), the results were not statistically significant (Fig. 2).

### Ki-67 expression in non-recurring and recurring leukoplakia

Nuclear Ki-67-expressing cells were detected mainly in the epithelium, and occasionally in inflammatory cells in the connective tissue (cells in the connective tissue were not included in the analysis) (Fig. 1). Although NOL were found to express lower levels of Ki-67 as compared to ROL (median: 8.0%, mean: 9%; range: 0–26 in NOL versus 10.0%, mean: 11.3% and range: 0–38 in ROL), the results were not statistically significant (Fig. 2).

### Univariable analyses for individual proteins and clinical data

**Figure 3:**
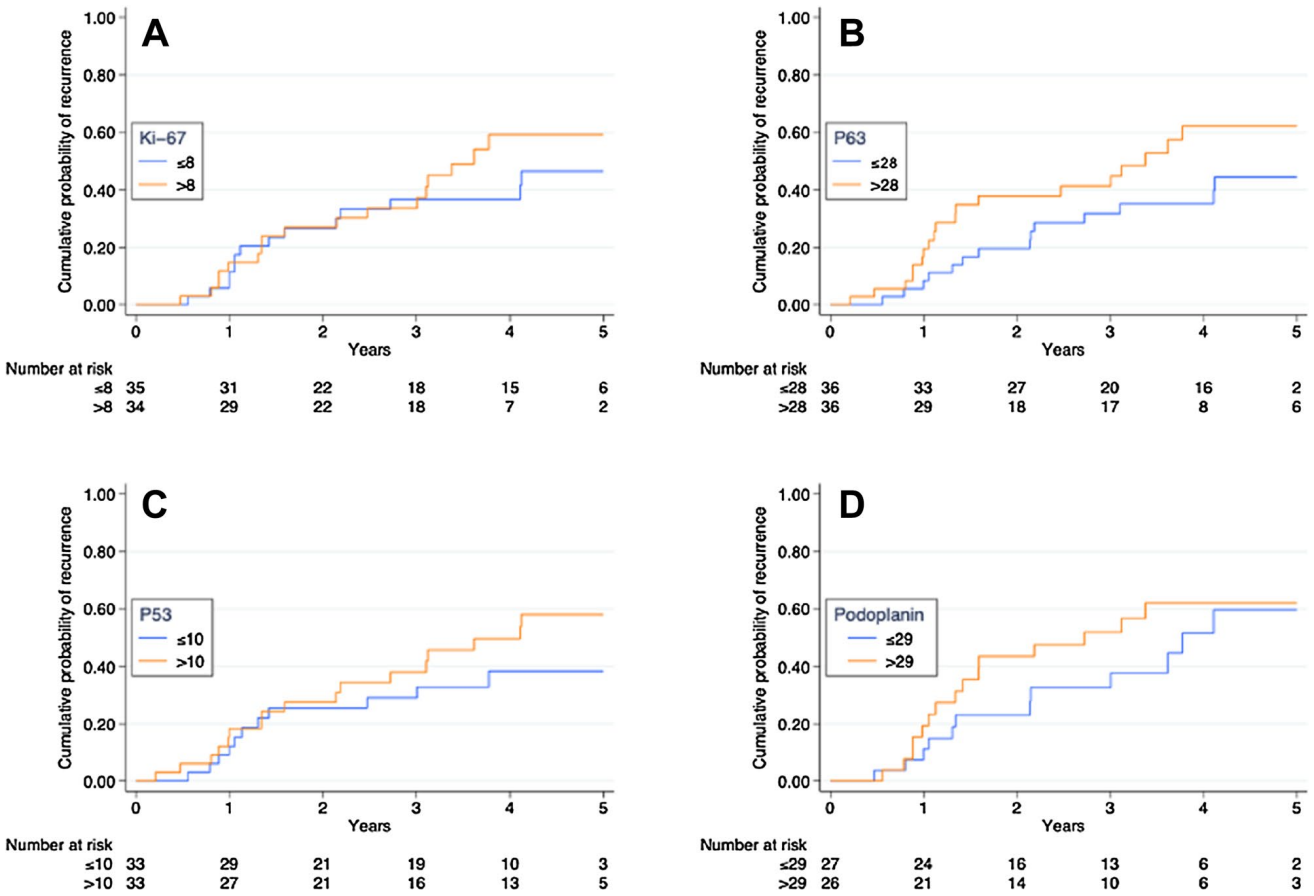
Kaplan–Meier analyses charting the cumulative probability of recurrence of leukoplakia divided by the median expression of Ki-67 (**A**), p63 (**B**), p53 (**C**) and podoplanin (**D**). Graphs show a trend that the high expression group of all four biomarkers by themselves are associated with recurrence after surgical excisions. SPSS Statistic (for Macintosh ver. 25.0 software package) was used to create the figure.

OL were divided into high and low expression groups using median value of the respective markers as cut-off points. Kaplan–Meier analyses charting the cumulative probability of recurrence of leukoplakia showed a trend that the high expression group of all four biomarkers by itself was associated with recurrence after surgical excisions (Ki-67; p = 0.72, p53; *p* = 0.27, p63; *p* = 0.11 and PDPN; *p* = 0.23; Fig. 3).

In univariable analysis, expression of p53, Ki-67, p53 and PDPN was analysed as continuous variables. Expression of p63 was significantly associated with a higher risk of recurrence after surgical excision (*p* = 0.047, HR (95% CI) = 1.02 (1.00–1.04); Table 2). Expression of Ki-67, p53 and PDPN was not significantly associated with a higher risk of recurrence after surgical excision (*p* = 0.085; *p* = 0.17; *p* = 0.25, respectively) (Table 2). The clinical factors that showed a significant association with an increased risk of recurrence after surgical excision were clinical diagnosis [*p* = 0.015, HR (95% CI) = 2.37 (1.19–4.73)] and the use of snuff [*p* = 0.005, HR (95% CI) = 3.64 (1.47–9.02)] (Table 2). Neither cell dysplasia, lesion size or multiple lesions were associated with an increased risk of recurrence (Table 2).

**Table 2:** Cox regression analyses based on expression of biomarkers as continuous variables and clinical and pathological factors for the recurrence of leukoplakia.

	Uni-variable analysis		Multi-variable analysis	
	HR (95% CI)	*p* value	HR (95% CI)	*p* value
**Clinical diagnosis**				
Homogeneous	1.00		1.00	
Non-homogeneous	2.37 (1.19–4.73)	**0.015**	2.06 (1.03–4.13)	**0.042**
**Size**				
< 200 mm^2^	1.00			
≥ 200 mm^2^	1.76 (0.88–3.53)	0.11		
**Dysplasia**				
No	1.00			
Yes	1.64 (0.74–3.64)	0.22		
**Number of lesions**				
Single	1.00			
Multiple	1.64 (0.79–3.38)	0.18		
**Smoker**				
Yes	0.80 (0.31–2.07)	0.64		
No	1.00			
**Snuff use**				
Yes	3.64 (1.47–9.02)	**0.005**	3.72 (1.47–9.42)	**0.006**
No	1.00		1.00	
Ki-67 expression	1.04 (1.00–1.09)	0.085		
p53 expression	1.01 (1.00–1.03)	0.17		
p63 expression	1.02 (1.00–1.04)	**0.047**	1.02 (1.00–1.04)	**0.035**
PDPN expression	1.01 (0.99 -1.03)	0.25		

Univariable cox analysis showed p63 expression, use of snuff and clinical diagnosis to be significantly associated with 5-year recurrence (*p* < 0.05). Multivariable Cox regression analysis demonstrated that p63 expression was an independent prognostic factor for the recurrence in leukoplakia patients. HR 1.02 for the p63 expression demonstrated an increased risk of recurrence with 2% (CI 0–4%) for each percentage increase in p63 expression (*p* < 0.05).

### Multi-variable analysis for individual proteins and clinical data

Multivariable analysis was performed to explore the influence of confounders. The results from this analysis, including biomarkers and clinicopathological factors that presented with a significant difference between the groups, revealed that expression of p63, the clinical diagnosis and the use of snuff were independent predictors for recurrence (*p* = 0.035, HR (95% CI) 1.02 (1.00–1.04); *p* = 0.042, HR (95% CI) 2.06 (1.03–4.13); *p* = 0.006, HR (95% CI) 1.02 (1.00–1.04), respectively) (Table 2).

### Combination of p53 and p63 expression and association with recurrence of OL

Patients were stratified into two different groups based on the combined expression of p53 and p63 using median expression score as cut-off values. An OL was considered in the high expression group if the expression of p53 and p63 was above the median cut-off values of both biomarkers. The rest of the lesions (with low expression of both markers, and high for p53 and low for p63 or vice versa) were considered low expression group. A total of 49 samples were in the low expression group and 16 samples in the high expression group. Kaplan–Meier curves were created for recurrence-free survival (RFS) and the log-rank test was used to compare RFS between the high and the low expression group.

**Figure 4:**
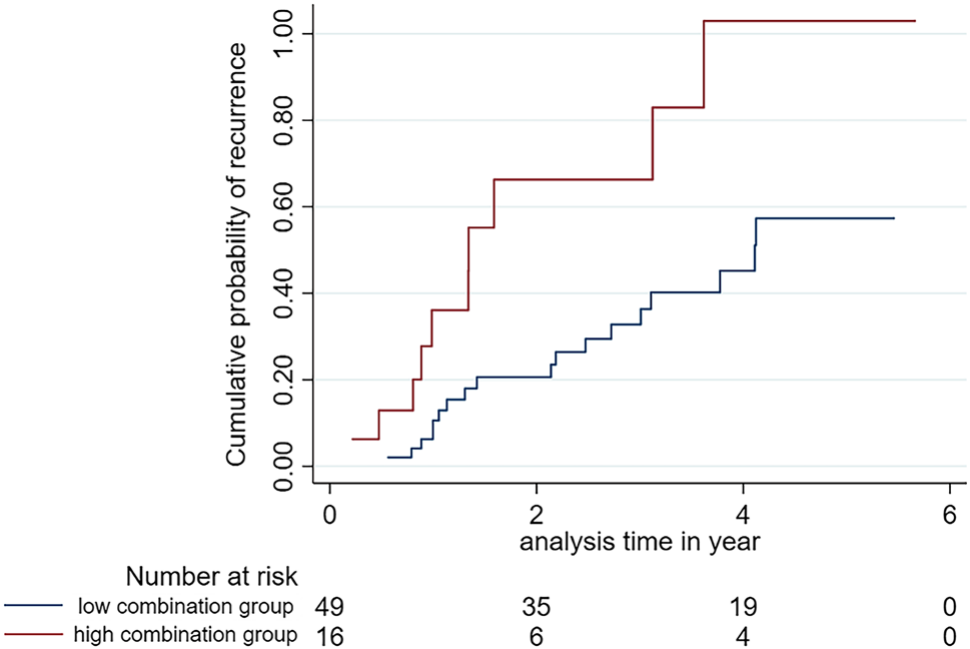
Kaplan–Meier analysis showing the cumulative probability of recurrence of leukoplakia. Leukoplakia were divided into high and low expression groups using the combined expression of p53 and p63 as described in the method section. The high expression group was found to have more recurrence hazards as compared to the low expression group (Log Rank, (*p* = 0.036). Stata Statistical Software (release 16) was used to create the figure.

The log-rank test showed a significant difference in RFS between high expression (p53 + p63) group and low expression group (*p* = 0.036) (Fig. 4). Cox regression analysis demonstrated that the combined expression of p63^high^ + p53^high^ was a prognostic factor for the recurrence in OL patients. An OL with a combined expression of p53^high^ and p63^high^ was associated with 2.24-times increase in risk of recurrence after surgical excision compared with an OL with a low expression of the same biomarkers (95% CI, 1.03–4.87; *p* = 0.041). When the HR was adjusted for the use of snuff the HR was 2.48 (95% CI, 1.13–5.44; *p* = 0.024) (Table 3).

**Table 3:** Cox regression analyses based on the combined expression of p63 and p53.

	Uni-variable analysis		Multi-variable analysis	
	HR (95% CI)	*p* value	HR (95% CI)	*p* value
**Snuff use**				
Yes	3.64 (1.47–9.02)	0.005	4.54 (1.49–13.83)	0.008
No	1.00		1.00	
Low p53 + p63 expression	1.00		1.00	
High p53 + p63 expression	2.24 (1.03–4.87)	0.041	2.48 (1.13–5.44)	0.024

Assumptions of proportional hazards were tested using Schoenfeld residuals. The global test showed no deviation from proportionality. Hence, we found no evidence that our specification for this multivariate cox regression model violated the proportional-hazards assumption (supplementary Table 1, supplementary Figure S3). Furthermore, comparison of Kaplan–Meier and Cox survivor functions for this model supported the same result (supplementary Figure S4).

## Discussion

The main finding in the present study is that the combined expression of p63 and p53 significantly correlated with the risk of recurrence of OL. Forty-five percent out of the total 73 OL that were totally excised recurred. This is in line with our earlier published results for the entire ORA-LEU-CAN study, where we found the total cumulative incidence of recurrence after 4 years to be 45%^4^.

The need for robust biomarkers to risk-stratify OL is evident. Genetic aberrations causing disturbances in cell cycle control is a key factor in cancer development^32^. Therefore, investigation of molecules involved in cell cycle control has been extensively addressed in OL and subsequent cancer transformation^11^. This study focused on investigations of molecules related to cell cycle control and recurrence of OL after surgical treatment. Since recurrence is a risk indicator for cancer development^4^, identification of biomarkers predicting recurrence may also be used in assessing cancer risk.

Two of the biomarkers studied in the present project -p53 and p63- are profoundly associated with cell cycle control and cancer development^32^. p53 is a key player in cell cycle control with the main function to shut down cells that are starting to or have lost proliferation control^33^. In normal conditions wild-type p53 is detectable in very low levels in oral epithelium, due to its negative feedback loop^34^. A mutated form of p53 has an extended half-life and can therefore be detected in higher numbers in pathologically altered epithelium^35^. Hence, an overexpression of p53 might indicate a mutation in the *TP53* gene. In OL, mutated *TP53* and loss of heterozygosity (LOH) at chromosome 9p as single markers and in combination, were risk factors for cancer^36^. Mutations in the *TP53* gene are also often found in OSCC^20^. In our patient cohort, ROL showed a significant increase in the expression level of p53 in comparison to NOL. These results indicate that loss of p53 mediated cell cycle control might be one of the molecular events in recurrence of OL.

Another key regulator of cell cycle, the p63 is closely related to p53^37^. In normal oral epithelium, p63 is present in basal cell layers of healthy oral epithelium, while in dysplasia p63 staining is not restricted to the basal cell layers^21^. The *TP63* gene is located on chromosome 3q27–29 and the p63 protein is involved in cell cycle arrest, apoptosis, and cell differentiation^37^. The *TP63* gene has two promoters resulting in two types of protein, TAp63 and DNp63, the latter also known as p40. The TAp63 group contains an N-terminal transactivation domain and has functions similar to p53. The DNp63 group lacks the transactivation domain and acts by inhibiting both p53 and TA p63, leading to cell proliferation^38,39^. In the current work, ROL showed a trend for higher expression of p63 as compared to NOL. Of note, univariate and multivariate Cox-regression analyses showed that higher expression of p63 could be an independent prognostic factor for recurrence in OL. Recurrence risk increased with 2% for each percentage increase in p63 expression. Nevertheless, a HR of 1.02 suggests that p63 alone might not be a strong predictor of recurrence in OL. This led us to investigate the prognostic significance of p63 in combination with p53.

OL with increased expression of both p53 and p63 showed a significantly higher risk of recurrence as compared to the rest of the lesions. Of note, the combination of p53 and p63 was found to be an independent prognostic factor with multivariate Cox analysis.

PDPN, a glycoprotein ubiquitously expressed throughout the body^23^, has been correlated with malignant transformation potential of OL^25,26^. In OL, PDPN has been proposed as a biomarker for increased cancer risk and recently, Aiswarya et al.^40^ showed that PDPN expression gradually increased with grade of dysplasia and early OSCC. Increased PDPN expression was shown to correlate with epidermal hyperproliferation in wound healing^41^. This fact indicates a possible influence of PDPN in wound healing and eventually also recurrence after surgical removal of OL. But our results show that PDPN expression was not correlated to recurrence.

Ki-67 as a marker for cell proliferation is used in histopathological evaluation of patients with both premalignant and malignant diseases. Expression pattern and intensity of Ki-67 have been correlated to grade of dysplasia in OL and cancer transformation^11,13^. Here, despite a trend for upregulation in ROL, the Ki67 was not found to correlate with recurrence potential of OL.

Several studies have proposed sets of biomarkers for predicting cancer transformation^42^. However, studies assessing biomarkers related to recurrence after surgery in a well-documented patient material are sparse. A strength of this study is the prospective design. However, the relatively small sample size, especially during analysis of combination markers can be considered as a limitation. Also, delineation of p53 and p63 isoforms could possibly have revealed information of interest. To some extent, lack of these data limits interpretation of the results.

In conclusion, a combined expression of p53 and p63 might be used as an independent prognostic marker for recurrence of OL after surgical removal. Since recurrence indicates an increased risk for cancer transformation, combining these biomarkers may be useful in identifying patients with an increased cancer risk.

## Patients and methods

In a prospective, on-going, longitudinal, multi-centre study (ORA-LEU-CAN Study) conducted in Sweden, patients with OL are included and followed up for 5 years. The study started in 2011 and initial results have previously been published^4^. In the present study, patients included during the period of 2011–2018 were subjected to analysis. The inclusion criterion was a clinically verified OL that was surgically removed with margin.

Clinical data, photographs and results of histopathological examinations were collected from the ORA-LEU-CAN database. Epithelial dysplasia was histopathologically scored according to the World Health Organization classification scale^43^. For the presence of dysplasia, a binary dysplasia scale was used, i.e., no dysplasia or dysplasia. The clinical diagnoses were re-reviewed by two specialists in oral medicine. When there was a difference of opinion regarding the diagnosis, a discussion was held until consensus was reached. A patient needed to be followed for at least 6 months to be included in the present study.

OL were removed with at least 2-mm clinical margin using conventional scalpel surgery and sent for histopathological analysis. Recurrence was defined as the reappearance of an OL at the site of surgery. A clinical healthy mucosa had to be recorded between the time of surgery and recurrence. The first 80 patients in the ORA-LEU-CAN study with OL that were excised in total^4^, were included consecutively between April 2011 and February 2016. In seven patients, tissue specimens were not possible to requisite. Hence, 73 patients proceeded to the analysis. The patients’ characteristics are listed in Table 1.

Patients were given written and verbal information about the study’s objectives. Both a written and a verbal consent were obtained from the patients at the clinic before inclusion. The written consent was signed by the patient and the clinician that included the patient in the study. The study was approved by the Regional Ethical Review Board in Gothenburg, Sweden (Dnr. 673–10) and was conducted in accordance with the Helsinki Declaration.

### Immunohistochemistry

4 μm-thick sections were prepared from formalin-fixed and paraffin-embedded (FFPE) tissue samples and placed onto positively charged glass slides (Flex IHC Microscope Slides; DAKO, Glostrup, Denmark). Immunohistochemical staining was performed with DakoEnVision™ + Dual Link System-HRP (Dako, Glostrup, Denmark) according to manufacturer instructions.

4 μm-thick sections were deparaffinized by placing the sections in xylene (p53, p63 and Ki-67 protocols) or by placing the sections in an incubator at 56 °C overnight followed by placing them in xylene (PDPN protocol). This was followed by rehydrating the sections in decreasing concentrations of ethanol and washing the sections in distilled water.

Antigen retrieval was performed by placing the sections in citrate buffer (pH 6.0; p63 protocol) or Tris–EDTA buffer, (pH 9.0; Cat. no: S2367, DAKO; p53, PDPN and Ki-67 protocols) in a pressure cooker at 100 °C for 15 min and 90 °C for 10 s.

After the sections had cooled to room temperature and been washed in tap water, endogenous peroxidase activity was blocked by hydrogen peroxide blocking solution. The sections were then washed with Tris-buffered saline + 0.1% Tween (TBST) and blocked with 10% normal goat serum in 3% bovine serum albumin (BSA). The sections were then incubated with the primary antibody (Table 4) for 1 h at room temperature. The anti-p53 antibody (M700101-2, Clone DO-7, DAKO) has been shown to detect both wild type and mutated versions of p53^44,45^. Analysis of the immunogen sequence used for the anti-p63 antibody (HPA006288, Atlas antibodies) indicated that the HPA006288 could detect both TAp63 and DNp63 isoforms (data not shown). The sections were washed with TBST. For p53 protocol the washing step was followed by inactivation of peroxidase by incubating the sections with DAKO REAL (code S2023, DAKO) peroxidase blocking solution. For p53, PDPN and Ki-67 protocols, sections were then incubated with anti-mouse secondary antibody conjugated with horseradish peroxidase labeled polymer (EnVision System, DAKO) for 30 min. For the p63 protocol the section were incubated with anti-rabbit secondary antibody conjugated with horseradish peroxidase labelled polymer (EnVision System, DAKO). Presence of antigen was visualized by staining with 3, 3′-diaminobenzidine (DAB, DAKO) followed by counterstaining with hematoxylin (DAKO) and mounting with EUKITT mounting medium. Sections from tonsil, OSCC and OL samples served as positive controls, while omission of the primary antibodies served as negative controls.

**Table 4:** Clone and dilutions of antibodies.

Antibodies and clones	Dilutions
PDPN (D2-40)—M361901-2, clone D2-40, DAKO	1:150
Monoclonal mouse anti-human p53 primary antibody—M7001*01-2,* Clone DO*-*7, DAKO	1:1000
Polyclonal rabbit antibody anti-human p63 primary antibody—HPA 006288, ATLAS ANTIBODIES, Sweden	1:100
Monoclonal mouse anti-human Ki-67—Clone MIB-1, DAKO	1:150

### Image data acquisition

All immuno-stained slides were scanned using NanoZoomer XR digital scanner, Hamamatsu scanner at 40X magnification and analyzed digitally in *Qupath* software (version 0.2.0-m4).

### Analysis of p53, p63, Ki-67 and podoplanin expression

Expression of p53, p63 and Ki-67 in ROL and in NOL were recorded and evaluated using *QuPath*: Open source software for digital pathology image analysis^46^. Analysis of antigen expression was blinded, and clinical information was revealed after cell counts were completed. Each section of the slide was divided into five parts of the epithelium, three in the centre (which were randomly chosen) and one in each resection margin (Figure S1). Area selected was 50,000–100,000 µm^2^ with a minimum of 200 cells included for each region. Cases where a clear resection margin was not possible to identify or resection margin was associated with artefacts, such as folding, tissue-tear or background noise, only the center parts were analysed. If there was an artefact within the tissue sample, the closest part of the artefact was analysed. To avoid risk of misleading results the stratum corneum was excluded from the analysis (Figure S1).

Before proceeding with digital scoring, the automatic analysis method was validated by comparing it with the semi-quantitative scoring using scanned slides in *QuPath* software. Ten different tissue samples were randomly selected. Manual calibration was done using the annotation counting tool. Automatic analysis was done using positive cell detection tool. Default specification in positive cell detection tool was modified selecting optical density sum and background radius was kept at 4 µm (Figure S2). Both automated and visual scoring were documented and compared. Afterwards, a comparison with three different trained pathologists (BT, DS, JÖ) was done and the default calibration was then evaluated. This specification was optimal and hence kept fixed for all other samples (Figure S1).

Expression of PDPN in ROL and NOL was recorded and evaluated visually with a light microscope by an experienced pathologist (BT). Brown granular membranous staining of the epithelial cells was considered positive for PDPN. The percentage of PDPN positive cells was defined by counting all cells on the basal layer, both negative and positive cells. In addition, arithmetical mean in each section was used to calculate the mean.

The expression of p53 was analysed for 66 samples. Seven samples were excluded because they were either torn, folded, or the epithelium was too small to analyse. For p63, 72 samples were analysed, and one was excluded due to artefacts in the epithelium. Sixty-nine samples with PDPN were analysed and four samples were excluded due to insufficient material. For Ki-67, 70 samples were included in the analysis and three samples had a ruptured epithelium and were therefore excluded.

### Statistical analysis

Recurrence versus no recurrence of OL was the primary outcome in our analysis. The follow-up time was defined as the time from the first excision surgery to the time of recurrence or to the last visit within the study protocol, which was maximum 5 years. In case of death before a recurrence, the death was censored (this was the case for one patient). A recurrence was defined as an event. Kaplan–Meier estimates were used to construct diagrams with cumulative incidence of OL stratified by the expression of individual as well as combined biomarkers, with a cut-off above or below the median value of the expression. To identify risk factors for predicting the recurrence of OL, uni- and multivariable Cox regression analyses were used based on expression of biomarkers as continuous variables and dichotomised clinical factors. The effect of analysed factors was described as a Hazard Ratio (HR) with 95% confidence interval (CI). Assumptions of proportional hazards were tested using Schoenfeld residuals. All variables in the Cox-regressions fulfilled the assumption. A *p* value < 0.05 was considered statistically significant.

Statistical analyses were carried out using the SPSS Statistic for Macintosh ver. 25.0 software package (IBM Corp., Armonk, NY) and StataCorp. 2019. Stata Statistical Software: Release 16. College Station, TX: StataCorp LLC.

## Supplementary Information

Supplementary Information.
