# Supplementary material for: Expression of p53, p63, podoplanin and Ki-67 in recurring versus non-recurring oral leukoplakia
Source: Sci Rep. 2021 Oct 21;11:20781. doi: 10.1038/s41598-021-99326-5 (PMC8531318; doi:10.1038/s41598-021-99326-5)
Supplement: Supplementary file 1 — Supplementary Information. [file 41598_2021_99326_MOESM1_ESM.docx]

# **Supplementary material**

***Supplementary Figure S1****: Digital cell quantification of p53, p63 and Ki67 using Qupath software.*

*A: A section divided into five parts of the epithelium, three in the centre and one in each resection margin. Area selected was 50 000 to 100 000 µm^2^ with minimum 200 cells included for each region.*

*B + C; illustrate the optimization process of the Qupath software. B shows the detected cells, both positive and negative cells and C illustrate the same area without detected cells.*


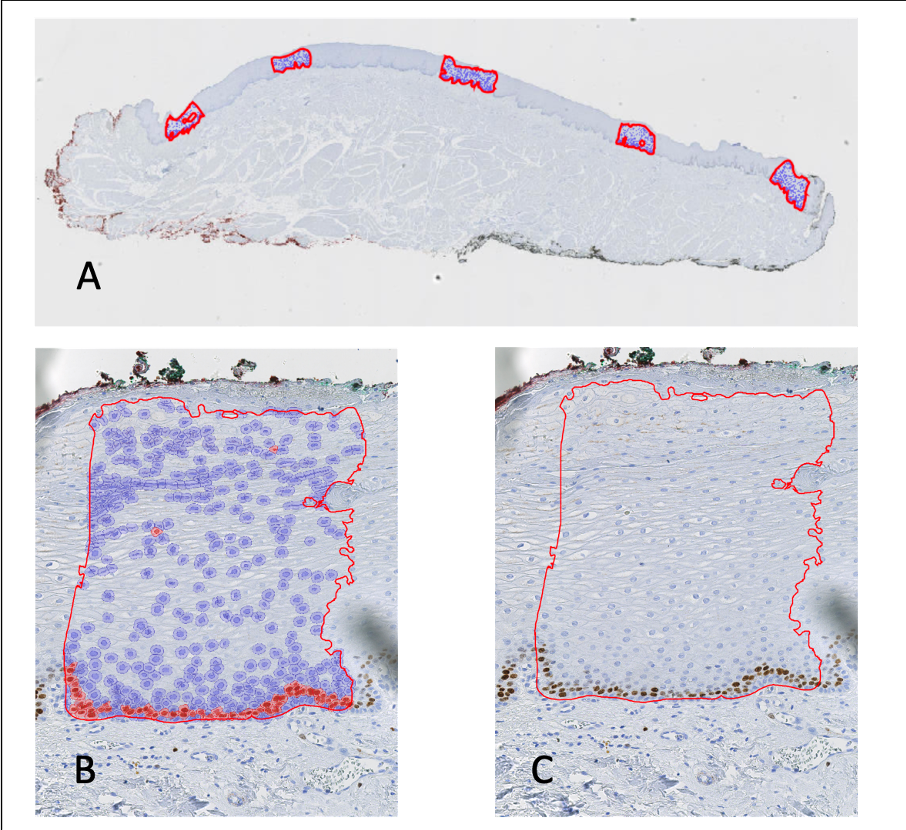


***Supplementary Figure S2.*** *Summarizing the digital methods used for IHC scoring for p53, p63 and Ki67 using Qupath software..*

*
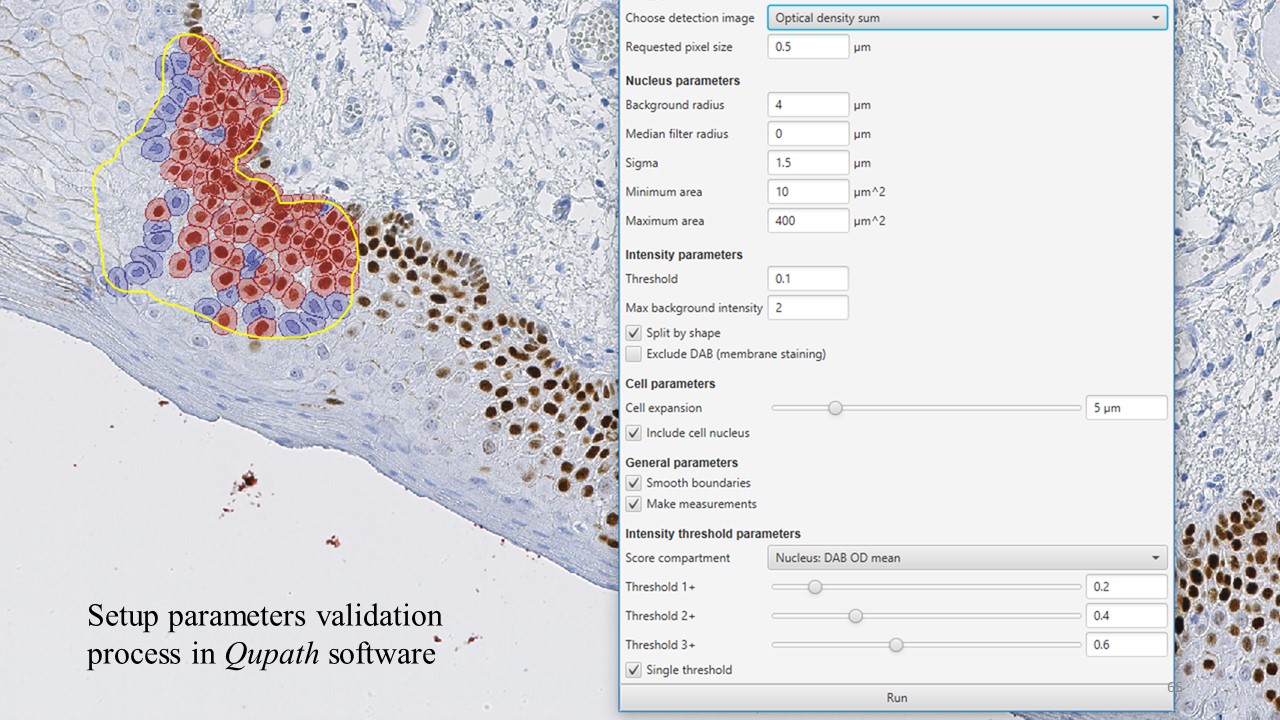
*

***Supplementary Figure S3****: Graphical representation of test of the proportional-hazard assumption. The curves are roughly parallel and not crossing, providing evidence in favor of the proportional-hazard assumption for p53 + p63 expression, controlling for covariant snuff use.*


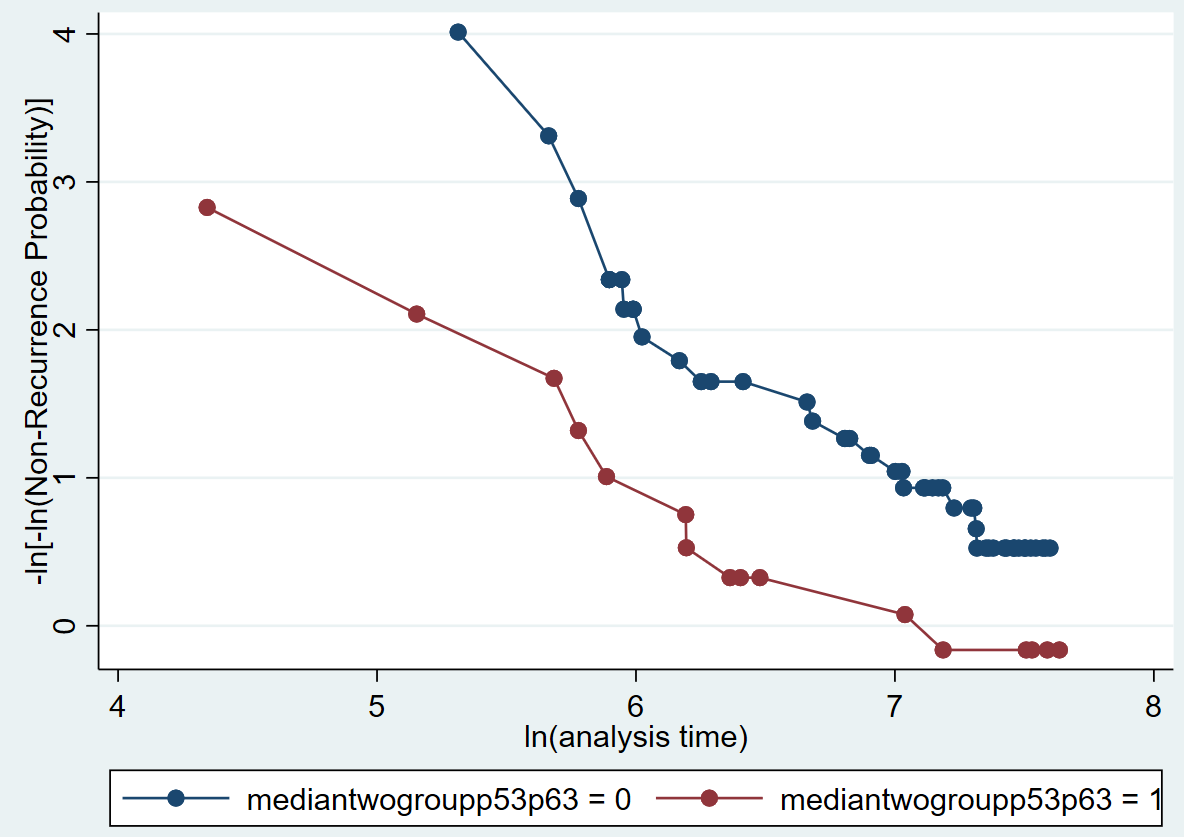


***Supplementary Figure S4.*** *Comparison of Kaplan-Meier and Cox survivor functions for combination marker (p53+p63). The graph showed that the observed values and predicted values were close together implying that the proportional-hazard assumption for combined expression of p53+p63 has not been violated.*

*.*

**
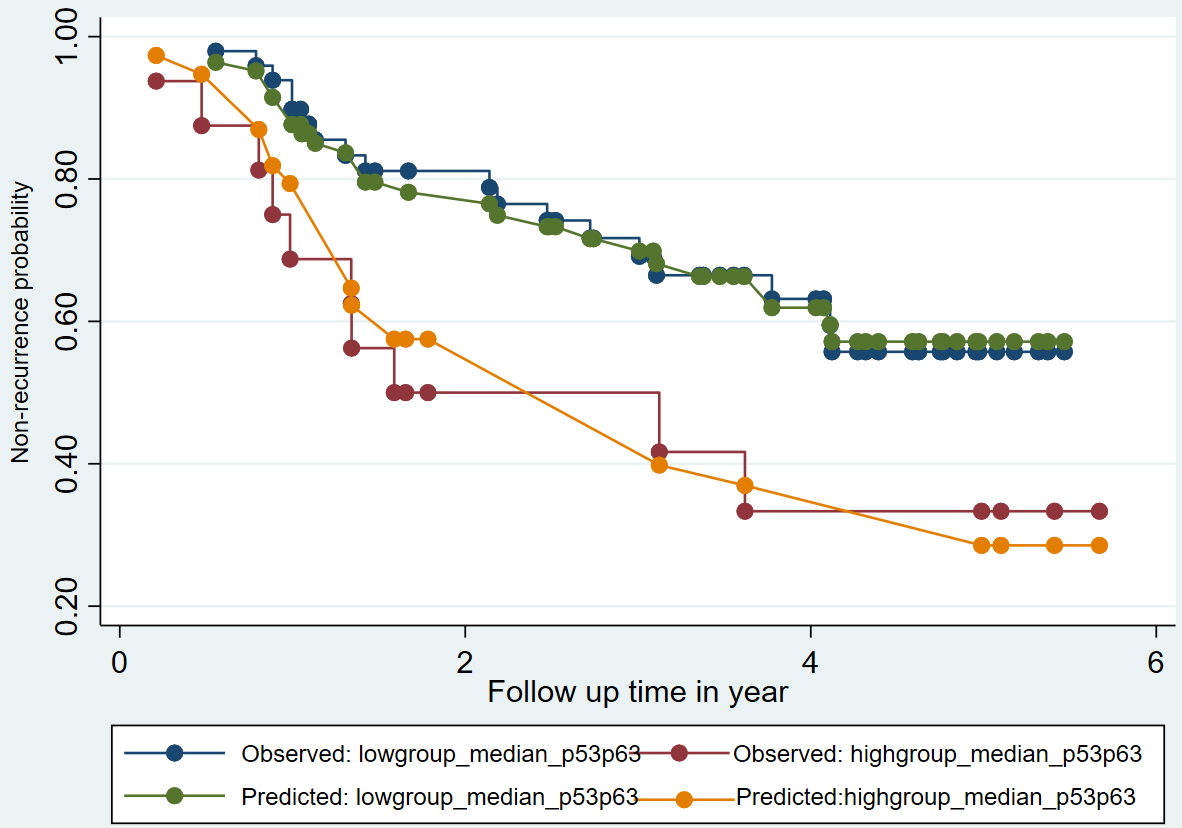
**

***Supplementary Table S1****: Global test for proportional-hazards assumption (specification). The variable-by-variable tests along with the overall test showed p-value is not significant (p>0.05) indicating no evidence that the specification violates the proportional-hazards assumption.*

|  | **rho** | **chi2** | **df** | **Prob>chi2** |
| --- | --- | --- | --- | --- |
| **Combine expression (p53+p63)**  **Snuff use** | -0.19011  0.18549 | 0.95  0.96 | 1  1 | 0.3291  0.3270 |
| **Global test** |  | 2.29 | 2 | 0.3185 |
